# Elevated plasma concentrations of lipoprotein (a) are associated with cardiovascular diseases in patients with early-onset type 2 diabetes mellitus

**DOI:** 10.3389/fendo.2025.1434745

**Published:** 2025-04-30

**Authors:** Juan Zhang, Jingjing Sang, Yanyan Jiang, Yi Zheng, Jing Zhang, Xuesen Liu, Huafeng Qiu, Kaijian Zhao, Hongmei Sun, Yang Yang, Hao Chen, Lei Yang

**Affiliations:** ^1^ Department of Scientific Research Section, The First People’s Hospital of Zhumadian, Affiliated Hospital of Huanghuai University, Zhumadian, China; ^2^ Institute of Monogenic Disease, School of Medicine, Huanghuai University, Zhumadian, China; ^3^ Department of Geriatric Endocrinology, The First Affiliated Hospital of Zhengzhou University, Zhengzhou, China; ^4^ Department of Cardiology, Zhumadian Central Hospital, Affiliated Hospital of Huanghuai University, Zhumadian, China; ^5^ School of Biological and Food Processing Engineering, Huanghuai University, Zhumadian, China; ^6^ Department of Scientific Research Section, Zhumadian Central Hospital, Affiliated Hospital of Huanghuai University, Zhumadian, China; ^7^ School of Medicine, Zhumadian Key Laboratory of Chronic Disease Research and Translational Medicine, Huanghuai University, Zhumadian, China

**Keywords:** lipoprotein(a), coronary heart disease, cerebrovascular disease, microvascular complications, early-onset type 2 diabetes mellitus

## Abstract

**Objective:**

To ascertain whether vascular complications and high lipoprotein (a) [Lp(a)] concentrations are related in individuals with early-onset type 2 diabetes mellitus (T2DM).

**Methods:**

This observational cross-sectional study included 591 individuals with early-onset T2DM who were divided into four groups based on Lp(a) values which was measured using immunoturbidimetry and presented as mg/dL: high, >50; intermediate, 30≤Lp(a)<50; low, 10≤Lp(a)<30; and very low, <10. The relationship between the risk of vascular complications and Lp(a) level was examined using a logistic regression model.

**Results:**

The median age of onset for individuals with early-onset T2DM (n=591) was 37 years, duration of diabetes was 12 years, and glycated hemoglobin (HbA1c) level was 8.8%. The median Lp(a) was 10.40 (4.80-21.80) mg/dL, and Lp(a) concentration did not correlate with age, sex, or glycemic control (*P*>0.05). Individuals in the low Lp(a) (OR=2.12, 95% CI 1.17-3.84, *P*<0.05), intermediate Lp(a) (OR=2.76, 95% CI 1.10-6.98, *P*<0.05) and high Lp(a) (OR=4.79, 95% CI 2.03-11.31, *P*<0.01) groups had an increased risk of coronary heart disease (CHD) compared with those in the very low Lp(a) group after adjustment. Nevertheless, among individuals with early-onset T2DM, there was no correlation between Lp(a) concentration and the risk of cerebrovascular disease (CVL) and microvascular complications (*P*>0.05).

**Conclusions:**

In patients with early-onset T2DM, Lp(a) concentration was independently associated with CHD. Lp(a) testing is essential to determine who has a latent high risk of CHD among patients with early-onset T2DM.

## Introduction

Lipoprotein (a) [Lp(a)] is a particle that resembles low-density lipoprotein (LDL) containing apolipoprotein B100 (apoB100) covalently attached to highly polymorphic glycoprotein, apolipoprotein(a) [apo(a)] (1). The interethnic variation in Lp(a) levels may be caused by the size of apo(a) and LPA genetic variation (2). Owing to the potent capacity of Lp(a) to transport oxidized phospholipids and its structural similarity to plasminogen, the cholesterol ester-rich Lp(a) is proposed to have stronger proatherogenic properties than LDL-cholesterol (LDL-C) (3, 4). Strong evidences from cross-sectional, prospective cohort, and genetic studies have confirmed that regardless of ethnicity, even at low levels of LDL-C, elevated Lp(a) levels have been identified as a risk factor for cardiovascular disease (CVD) (5–9). Elevated levels of Lp(a) have also been linked to a higher risk of ischemic stroke and poor functional outcomes, based on recent research (10–12). Additionally, at low inflammatory or LDL-C levels, the risk of stroke recurrence associated with high Lp(a) levels is reduced (13).

Limited data regarding Lp(a) in type 1 diabetes mellitus (T1DM) have been reported. Studies have shown that patients with well-controlled T1DM have low Lp(a) levels to T1DM patients with poorer metabolic control, and Lp(a) is regarded as an important risk factor for CVD, albuminuria, and calcified aortic valve disease in individuals with T1DM (14, 15). Lp(a) in type 2 diabetes mellitus (T2DM) presents a significantly more complex scenario. Although plasma Lp(a) levels have been consistently reported to be lower in individuals with T2DM than in nondiabetic controls, compelling evidences suggest that higher circulating Lp (a) levels in individuals with T2DM are markedly related to higher odds of CVD and diabetic nephropathy and retinopathy (16–21). A reduced Lp(a) concentration in patients with T2DM and their increased risk of diabetic complications is called the Lp(a) paradox in T2DM (22). Gene variants associated with lipid and lipoprotein metabolism in individuals with T2DM may help explain their low Lp(a) levels. Consistently, reduced Lp(a), apolipoprotein (AII), and apolipoprotein (CIII) levels have been observed in patients with maturity-onset diabetes of the young (MODY) carrying the HNF4A (Q268X) mutation (23).

Based on recent epidemiological research, the age-standardized incidence of early-onset T2DM has increased remarkably from 117.22 per 100,000 in 1990 to 183.36 per 100,000 in 2019 (24). Monogenic diabetes mainly occurs in patients with early-onset T2DM, with a high mutation detection frequency of 16% (25, 26). Mutations in HNF1A, HNF4A, and HNF1β genes involved in Lp(a) expression account for more than 50% of monogenic diabetes with a known genetic cause, which may lead to the complexity of concentrations of Lp(a) in patients with T2DM (22, 25, 27, 28).

The purpose of this cross-sectional study is to ascertain whether vascular complications of individuals with early-onset T2DM are related to high Lp(a) concentrations, and the association between Lp(a) levels and glycemic control. We tested the hypothesis that high levels of Lp(a) are associated with increased risk of cardiovascular diseases in patients with early-onset T2DM.

## Research design and methods

### Study population

This observational cross-sectional study recruited patients with early-onset T2DM from the Department of Endocrinology, Zhumadian Central Hospital and the First Affiliated Hospital of Zhengzhou University from June 2021 to June 2023. Early-onset T2DM refers to T2DM that is diagnosed in childhood or early adulthood (29). The participant inclusion criteria were (1) diagnosis of diabetes according to criteria set by the American Diabetes Association (2021) and (2) onset age ≤ 40 years (30). Patients with T1DM and secondary diabetes (such as diabetes induced by exocrine pancreas diseases, drugs, or chemicals) were excluded. Patients without Lp(a), glycated hemoglobin (HbA1c), and fasting plasma glucose (FPG) measurements were also excluded. Finally, 591 patients with early-onset T2DM were included, of which 20 individuals had monogenic diabetes of a known cause. Based on a prior report, individuals with early-onset T2DM were categorized into four groups according to Lp(a) levels: high Lp(a) (≥50 mg/dL), intermediate Lp(a) (≥30 to <50 mg/dL), low Lp(a) (≥10 to <30 mg/dL), and very low Lp(a) (<10 mg/dL) groups (18). This study adhered to the principles of the Declaration of Helsinki and was approved by the Ethics Committees of Zhumadian Central Hospital and the First Affiliated Hospital of Zhengzhou University. Written informed permission was obtained from each patient to take part in the research.

### Clinical features and laboratory parameters

Basic information on the study population, such as age, onset age of diabetes, height, weight, blood pressure, smoking history, family history of diabetes, diabetic complications, and hypoglycemic treatment, was obtained from electronic medical records. Various blood samples were collected from patients for laboratory analysis after overnight fasting for 10-12 hours. Using immunoturbidimetry, Lp(a) levels were determined and are presented as mg/dL. For calibration, the Lp(a) protein validation standard was used, and the coefficient of variation of the repeated measurements was less than 10%. An automatic biochemistry analyzer (7600-020; Hitachi, Tokyo, Japan) was used to measure LDL-C, triglyceride, total cholesterol, serum creatinine (SCr), blood urea nitrogen, and high-density lipoprotein cholesterol. Plasma glucose levels were measured using an enzymatic hexokinase method. HbA1c levels were determined using high-performance liquid chromatography (Bio-Rad, Hercules, CA, USA). Urinary protein quantification and urinary microalbumin levels were identified in 24-hour urine samples using immunoturbidimetry.

### Definitions of diabetic complications

As previously reported, macrovascular complications were defined based on the international Classification of diseases, 10th edition (ICD-10) (15). Macrovascular complications are composites of coronary heart disease (CHD) and cerebrovascular disease (CVL). CHD included angina pectoris (I20), myocardial infarction (I21, I25.2), coronary artery bypass graft history (Z95.1), and coronary angioplasty implant (Z95.5). CVL included ischemic stroke (I63.4, I63.5) and transient ischemic attack (TIA) (G45.9). The microvascular complications of diabetes were defined according to the ADA guidelines (31). Diabetic nephropathy (DN) was defined as the presence of macroalbuminuria [urinary albumin-creatinine ratio (UACR)≥300 mg/g)] or microalbuminuria (30≤UACR<300mg/g) and/or impaired renal function characterized by a decreased estimated glomerular filtration rate (eGFR). Retinopathy confirmed by fundus photography of the retina was defined as diabetic retinopathy (DR). Diabetic peripheral neuropathy (DPN) was diagnosed by a clinician based on the patient’s symptoms and nerve conduction studies (NCS).

### Statistical analysis

SPSS 29.0 and GraphPad Prism 8 were used for statistical analyses and graphics production. Values are presented as median (interquartile range) or mean ± SD for continuous variables and as frequency (%) for categorical variables. The distribution pattern was tested using the Kolmogorov-Smirnov test. Student’s t-test was employed to compare the means of the differences between the groups, Mann-Whitney U test for medians, and Pearson χ^2^ tests for percentages, where appropriate. A logistic regression model was used to assess the correlation between macrovascular and microvascular problems and Lp(a) level. Statistical significance was defined as a *P* value <0.05.

## Results

### Clinical and biochemical characteristics of patients with early-onset T2DM

**Table 1** displays the characteristics of the 591 patients with early-onset T2DM. Compared with early-onset T2DM patients with an unknown cause, patients with monogenic diabetes had a younger onset age (19 vs. 37 years, *P*<0.01), shorter duration (1 vs. 13 years, *P*<0.01), and comparable HbA1c level (9.0 vs. 8.8%, *P*>0.05). The total prevalence of macrovascular complications in individuals with early-onset T2DM was 18.4%, including 13.7% with CHD and 6.9% with CVL. The total prevalence of microvascular complications was 58.2%, including 29.1% with DN, 34.3% with DR, and 28.8% with DPN. The range of Lp(a) values for those with early-onset T2DM was 10.40 (4.80-21.80) mg/dL, and comparable Lp(a) levels were detected in patients with monogenic diabetes and those with diabetes with an unknown cause [9.19 (3.30-15.76) vs. 10.4 (4.86-22.00) mg/dL, *P*>0.05] (**Table 1**).

**Table 1:** Clinical and biochemical characteristics of patients with early-onset T2DM.

	Early-onset T2DM (n=591)	Unknown cause of early-onset T2DM (n=571)	Monogenic diabetes (n=20)
Age (years)	51 (39-58)	51 (41-58)	22 (18-30)^**^
Onset age (years)	37 (31-39)	37 (32-39)	19 (15-24)^**^
Duration (years)	12.00 (6.00-20.00)	13.00 (7.00-20.00)	1.00 (0.02-4.75)^**^
BMI (kg/m^2^)	25.00 (22.86-27.36)	25.01 (22.86-27.42)	23.94 (20.60-25.43)^*^
SBP (mmHg)	130 (120-139)	130 (120-140)	117 (110-128)^*^
DBP (mmHg)	80 (70-85)	80 (70-85)	79 (73-87)
History of hypertension, n (%)	245 (41.5)	245 (42.9)	0
Smoking status			
Never, n (%)	378 (64.0)	363 (63.6)	15 (75)
Previous, n (%)	19 (3.2)	18 (3.2)	1 (5)
Current, n (%)	194 (32.8)	190 (33.3)	4 (20)
HbA1c (%)	8.8 (7.4-10.2)	8.8 (7.4-10.2)	9.0 (7.3-11.6)
FPG (mmol/L)	7.71 (6.26-9.67)	7.73 (6.27-9.71)	6.95 (5.78-8.13)
2hPG (mmol/L)	12.90 (9.93-15.96)	12.89 (9.89-15.89)	13.65 (10.70-18.95)
TG (mmol/L)	1.42 (0.98-2.25)	1.44 (0.99-2.28)	1.08 (0.73-1.34)^**^
TC (mmol/L)	4.62 (3.90-5.44)	4.62 (3.91-5.45)	4.37 (3.29-4.91)
LDL-C (mmol/L)	2.78 (2.07-3.45)	2.78 (2.09-3.49)	2.82 (2.03-3.23)
HDL-C (mmol/L)	1.02 (0.86-1.25)	1.02 (0.86-1.25)	1.05 (0.94-1.44)
Lp(a) (mg/dl)	10.40 (4.80-21.80)	10.40 (4.86-22.00)	9.19 (3.30-15.76)
BUN (mmol/L)	5.1 (4.2-6.3)	5.1 (4.2-6.3)	5.3 (4.2-5.9)
Creatinine (μmol/L)	62.00 (51.00-74.00)	62.00 (51.00-74.00)	53.00 (44.00-74.00)
Albuminuria (g)	0.09 (0.04-0.21)	0.08 (0.04-0.21)	0.13 (0.11-0.20)
Macrovascular complications			
CHD, n (%)	81 (13.7)	77 (13.5)	4 (20)
CVL, n (%)	41 (6.9)	39 (6.8)	2 (10)^**^
Microvascular complications			
Nephropathy, n (%)	172 (29.1)	167 (29.2)	5 (25)
Retinopathy, n (%)	203 (34.3)	196 (34.3)	7 (35)
Neuropathy, n (%)	170 (28.8)	163 (28.5)	7 (35)
Antidiabetic therapy			
Diet, n (%)	588 (99.5)	568 (99.5)	20 (100)
OHA, n (%)	399 (67.5)	383 (67.1)	16 (80)
Insulin, n (%)	406 (68.7)	399 (69.9)	7 (35)^**^

Data are presented as mean ± SD, the median (interquartile range), or n (%). CHD, coronary heart disease; CVL, cerebrovascular disease; OHA, oral hypoglycemic agents. Unknown cause of early-onset T2DM vs. Monogenic diabetes, ^*^ *P*<0.05, ^**^ *P*<0.01.

### Lp(a) distribution of patients with early-onset T2DM

**Figure 1:**
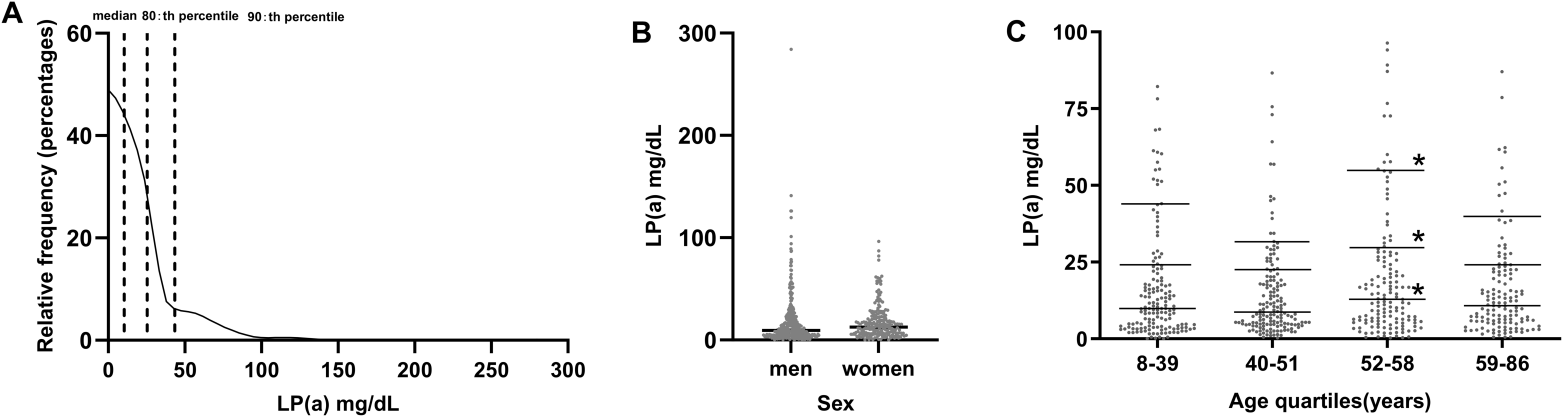
**(A)** Distribution of lipoprotein (a) [Lp(a)] levels in patients with early-onset T2DM (n=591). **(B)** Comparison of Lp(a) levels between men and women. **(C)** Comparison of Lp(a) levels between age quartiles. ^*^ *P*<0.05.

**Figure 1A** illustrates the Lp(a) skewed distribution in patients (n=591) with early-onset T2DM; the median, 80th, and 90th percentiles were 10.4, 25.48, and 43.36 mg/dL, respectively. There were no discernible variations in the concentration of Lp(a) between sexes (**Figure 1B**). Patients were grouped according to age quartile (Q1: 8-39; Q2: 40-51; Q3: 52-58; and Q4: 59-86 years). Compared with the lowest respective quartile, no significant differences in levels of Lp(a) across age quartile groups were found at the median (Q1: 9.9, Q2: 8.7, Q3: 12.9, Q4: 10.8), 80th percentile (Q1: 24.1, Q2: 22.58, Q3: 29.52, Q4: 24.34), or 90th percentile (Q1: 43.91, Q2: 31.30, Q3: 54.82, Q4: 39.86)] (*P*>0.05 for all comparisons except for Q3) (**Figure 1C**).

### Correlation between diabetic complications and Lp(a) levels in individuals with early-onset T2DM

In individuals with early-onset T2DM, binary logistic regression was used to examine the relationship between the risk of diabetic complications and plasma Lp(a) levels. In contrast to those in the very low Lp(a) group, individuals in the low (OR=1.91, 95% CI 1.11-3.29, *P*<0.05), intermediate (OR=3.04, 95% CI 1.30-7.08, *P*<0.05) and high (OR=3.28, 95% CI 1.53-7.06, *P*<0.01) Lp(a) groups had a higher CHD risk (**Table 2**). Lp(a) levels remained independently linked to a higher CHD risk after adjusting for confounding factors [low Lp(a) vs. very low Lp(a) group: OR=2.12, 95% CI 1.17-3.84, *P*<0.05; intermediate Lp(a) vs. very low Lp(a) group: OR=2.76, 95% CI 1.10-6.98, *P*<0.05; high Lp(a) vs. very low Lp(a) group: OR=4.79, 95% CI 2.03-11.31, *P*<0.01]. Nevertheless, independent of adjusting for confounding factors, Lp(a) levels in individuals with early-onset T2DM were not associated with the risk of microvascular complications and CVL (*P*>0.05).

**Table 2:** Association between Lp(a) levels and diabetic complications in patients with early-onset T2DM(n=591).

	All	Very low Lp(a)<10mg/dL(n=289)	Low 10≤Lp(a) <30 mg/dL (n=214)	Intermediate 30≤Lp(a) <50 mg/dL (n=39)	High Lp(a)≥50 mg/dL (n=49)
CHD, n (%)	81	26 (32.10)	34 (41.98)	9 (11.11)	12 (14.81)
Crude model, OR (95%CI)		1	1.91 (1.11-3.29)^*^	3.04 (1.30-7.08)^*^	3.28 (1.53-7.06)^**^
Adjusted model 1, OR (95%CI)		1	2.12 (1.17-3.84)^*^	2.76 (1.10-6.98)^*^	4.79 (2.03-11.31)^**^
CVL, n (%)	41	17 (41.46)	16 (39.02)	4 (9.76)	4 (9.76)
Crude model, OR (95%CI)		1	1.29 (0.64-2.62)	1.83 (0.58-5.74)	1.42 (0.46-4.42)
Adjusted model 2, OR (95%CI)		1	1.54 (0.70-3.39)	1.51 (0.41-5.51)	2.16 (0.61-7.67)
Nephropathy, n (%)	172	81 (47.09)	59 (34.30)	14 (8.14)	18 (10.47)
Crude model, OR (95%CI)		1	0.98 (0.66-1.45)	1.44 (0.71-2.90)	1.49 (0.79-2.81)
Adjusted model 3, OR (95%CI)		1	1.19 (0.76-1.86)	1.62 (0.73-3.60)	1.55 (0.77-3.12)
Retinopathy, n (%)	203	95 (46.80)	77 (37.93)	12 (5.91)	19 (9.36)
Crude model, OR (95%CI)		1	1.15 (0.79-1.66)	0.91 (0.44-1.87)	1.29 (0.69-2.42)
Adjusted model 4, OR (95%CI)		1	1.15 (0.77-1.70)	0.85 (0.40-1.79)	1.11 (0.58-2.15)
Neuropathy, n (%)	170	73 (42.94)	67 (39.41)	16 (9.41)	14 (8.24)
Crude model, OR (95%CI)		1	1.35 (0.91-2.00)	2.06 (1.03-4.11)	1.18 (0.60-2.32)
Adjusted model 5, OR (95%CI)		1	1.32 (0.85-2.05)	2.17 (0.96-4.87)	1.07 (0.51-2.25)

Adjusted model 1: adjusted for age, course of disease, history of hypertension, TC, LDL-C and BUN. Adjusted model 2: adjusted for age, course of disease, history of hypertension, TC, LDL-C and BUN. Adjusted model 3: adjusted for smoking status, age, course of disease, history of hypertension, BMI, TG, HDL-C, BUN and creatinine. Adjusted model 4: adjusted for age, course of disease, history of hypertension and BUN. Adjusted model 5: adjusted for age, course of disease, history of hypertension, BMI, LDL-C and BUN. CHD, coronary heart disease; CVL, cerebrovascular disease. ^*^ *P*<0.05, ^**^ *P*<0.01.

### Plasma Lp(a) level in relation to glycemic control

**Figure 2:**
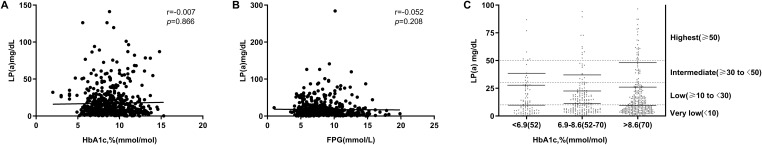
Association between glycemic control and Lp(a) levels. **(A, B)** Correlation of plasma lipoprotein (a) [Lp(a)] with glycated hemoglobin (HbA1c) and fasting plasma glucose (FPG) levels. **(C)** Absolute values of Lp(a) in each HbA1c group. Patients were divided into three groups according to HbA1c level categorized as representing good, <6.9% (n=89); intermediate, 6.9-8.6% (n =208); or poor, >8.6% (n=294) glycemic control.

As shown in **Figures 2A, B**, plasma Lp(a) levels in individuals with early-onset T2DM (n=591) were not correlated with HbA1c levels (r=-0.007, *P*=0.866) and FPG (r=-0.052, *P*=0.208). Patients were further separated into three groups according to HbA1c level categorized as representing good, <6.9% (n=89); intermediate, 6.9-8.6% (n =208); or poor, >8.6% (n=294) glycemic control, and Lp(a) levels remained uncorrelated with HbA1c levels, with median values at 9.76, 11.10, and 9.60 mg/dL, 80th percentile values at 27.80, 22.70, and 26.20 mg/dL, and 90th percentiles values at 38.50, 37.10, and 48.00 mg/dL, in the three groups, respectively (**Figure 2C**).

## Discussion

High Lp(a) is a lipid abnormality primarily caused by variation in the LPA gene, substantial evidence has linked high Lp(a) to an increased risk of CVD (32). Nevertheless, the contribution of Lp(a) to the microvascular and macrovascular consequences of diabetes is still debatable (22). This cross-sectional study demonstrated that the plasma levels of Lp(a) in individuals with early-onset T2DM were not influenced by sex, age, or glycemic control. Moreover, regardless of adjustment for confounders or not, the prevalence of CHD was markedly increased in the high and intermediate Lp(a) groups, while Lp(a) levels were not evidently linked to CVL or diabetic microvascular complications including nephropathy, retinopathy and neuropathy. Therefore, our research provides evidence that in patients with early-onset T2DM, Lp(a) elevation is a risk factor for CHD, regardless of glycemic management.

Although elevated Lp(a) levels are not causally associated with the risk of T2DM, multiple studies have demonstrated that elevated plasma levels of Lp(a) in individuals with T2DM are particularly at an increased risk of CVD (17, 22). In our early-onset T2DM cohort, patients in the very low Lp(a) group had a lower risk of CHD than those in the high and intermediate Lp(a) groups, providing more evidence that high levels of Lp(a) contribute to CVD. Furthermore, a recent study discovered a substantial increase in the likelihood of recurrent cardiovascular events with high levels of Lp(a) in patients with CHD (33). Compared with individuals with little Lp(a) exposure, individuals with T2DM with cumulative Lp(a) exposure are more likely to experience unfavorable cardiovascular outcomes (34). However, the effect of plasma Lp(a) concentration on CVL in patients with diabetes is not as convincing as that on CVD. Despite that raised Lp(a) levels have been reported to be linked to an elevated risk of ischemic stroke, unfavorable functional outcomes, and stroke recurrence, the relation of Lp(a) and CVL in the diabetes cohort was confusing (10–13, 15, 35, 36). In individuals with early-onset T2DM, increased Lp(a) levels did not increase the risk of CVL. Lp(a) concentration was not associated with CVL in patients with early-onset T2DM and T1DM, but was related to the increased risk of ischemic stroke and its unfavorable functional outcome in patients with T2DM, possibly due to the age of onset of diabetes in these individuals (the median of age at diagnosis for early-onset T2DM, T1DM and T2DM, 37 vs. 23 vs.51) (15, 35).

The function of elevated Lp(a) in microvascular problems remains debatable, despite the well-known association between CVD and Lp(a) in individuals with diabetes. Although decreased Lp(a) has previously been reported to be associated with increased microvascular damage, a meta-analysis of 11 observational studies involving 9304 individuals with T2DM indicated that increased Lp(a) in individuals with T2DM was independently related to a greater risk of DN (19, 37). Most epidemiological studies support high circulating Lp(a) concentrations as an independent risk factor for DR, although a correlation between retinopathy and Lp(a) has not been observed in some studies (20, 21). Recently, Shariatzadeh found that anti-inflammatory and anti-angiogenic capacity was affected in patients with T2DM and DR, supporting the unfavorable impact of high Lp(a) levels in DR (38). Unfortunately, we did not observe a relationship between the risk of microvascular disease and the concentration of Lp(a), including DN, DR, and DPN, in Han Chinese individuals with early-onset T2DM. This negative observation may be related to the small sample size and ethnic background in this study, as studies supporting the irrelevance between Lp(a) and microvascular complication also existed the phenomenon of small sample sizes and genetic heterogeneity (19, 21, 39). Additionally, from a genetic perspective, although the *LPA* single-nucleotide polymorphisms (SNPs) rs3798220 and rs10455872 affect the plasma levels of Lp(a), a prospective genetics-based analysis revealed no evident correlation between the development of microvascular problems in T2DM and *LPA* SNPs or Lp(a) concentration (40). However, the lack of association in Caucasian populations did not well support our results owing to ethnic heterogeneity. Further large cohort genetic studies conducted in Asian populations will help illuminate the correlation of Lp(a) with diabetic microvascular complications.

The plasma concentration of Lp(a) is genetically determined and remains almost constant throughout the life of an individual; thus, significant inter-individual as well as intra- and inter-ethnic differences are observed in the concentrations of Lp(a), but they are not influenced by age or sex (41). No discernible difference in plasma Lp(a) levels of early-onset T2DM patients was observed between the men and women, or among different age levels in our study. Additionally, Lp(a) concentrations showed no differences among patients with good, intermediate, or poor glycemic control, as indicated by HbA1c, suggesting that the improvement in glycemic control was not accompanied by a reduction in Lp(a). Previous research showing no correlation between Lp(a) levels and glycemic management in individuals with T2DM lends credence to our findings (22). However, Littmann found that metabolic control in patients with T1DM was associated with plasma levels of Lp(a) (15). This could be due to the effect of insulin on the production of Lp(a) in the liver and poor renal function in patients with T1DM. The levels of Lp(a) are mostly elevated in patients with T1DM owing to metabolic effects; nevertheless, publications have consistently reported that plasma Lp(a) levels are lower in patients with T2DM than in controls (22). Lp(a) levels in our early-onset T2DM cohort [10.40 (4.80-21.80) mg/dL] were also lower than that of controls with normal blood glucose regulation [15.2 (6.8–35.5) mg/dL] and patients with diabetes [14.7 (6.6–33.8) mg/dL] reported by Jin (18). The early-onset T2DM cohort included patients with monogenic diabetes resulting from mutations in the *HNF1β*, *HNF4A*, and *HNF1A* genes associated with Lp(a) expression, which may help explain the lower levels of Lp(a) (22, 25, 27, 28). Consistently, low Lp(a) levels [9.19 (3.30-15.76) mg/dL] were detected in patients with monogenic diabetes with these gene mutations (n=9). Furthermore, patients with MODY with the *HNF4A* (Q268X) mutation have decreased plasma Lp(a) concentrations (23).

The role of high Lp(a) levels in CHD and adverse cardiovascular outcomes in individuals with T2DM with cumulative Lp(a) exposure has prompted an ongoing search for strategies to reduce Lp(a) levels. Two randomized, double-blind trials covering 13 research centers found that in individuals with high levels of Lp(a), antisense oligonucleotides targeting apolipoprotein (a) were effective in lowering Lp(a) concentrations and Lp(a)-related cardiovascular risk (42). Subsequent investigations revealed that in people with CVD and increased Lp(a) levels, the hepatocyte-directed antisense oligonucleotide lowered the levels of Lp(a) in a dose-dependent manner (43). These results support the favorable impact of declining Lp(a) levels on cardiovascular risk in patients with high Lp(a) levels. In addition, Yu et al. recently found that evolocumab therapy brings beneficial changes to plaque components in patients with T2DM and attenuates pericoronary adipose tissue (PCAT) density, which may be caused by a decrease in Lp(a) (44). A worse prognosis and higher probability of cardiac mortality are associated with elevated PCAT density (45). Owing to their prolonged illness duration and early onset age, patients with early-onset T2DM are more prone to cardiovascular events (46). Moreover, individuals with early-onset diabetes have a significantly increased risk of heart failure, and excessive cardiorenal risk factors may be the main drivers of the progression of heart failure (47). Therefore, Lp(a) treatment for early-onset T2DM in patients with high Lp(a) concentrations may reduce long-term cardiovascular risk to some extent.

However, the current study had several limitations. Firstly, This is a cross-sectional study, and we cannot provide evidence for a causative role of Lp(a) for CHD in patients with early-onset T2DM. Secondly, it is also limited in sample size, especially the size of the intermediate and high Lp(a) groups, which may be the main reason that the irrelevance between Lp(a) and microvascular complication. Thirdly, we only incorporated 20 patients with monogenic diabetes, which is difficult to highlight the effect of mutations in HNF1β, HNF4A, and HNF1A genes associated with Lp(a) expression on Lp(a) concentration in patients with early-onset T2DM. In addition, the use of both immunoassay-based and mass spectrometry (MS)-based test for Lp(a) may be more convincing, since MS-based test expressed in molar units is the gold standard method for Lp(a) measurement.

In conclusion, in our cross-sectional study, increased Lp(a) levels were associated with an elevated risk of cardiovascular disease in patients with early-onset T2DM, independent of glycemic control. Future prospective studies with a larger sample size including more cases with monogenic diabetes can further elucidate the precise role of Lp(a) in the vascular complication of early-onset T2DM patients.

## Data Availability

The original contributions presented in the study are included in the article. Further inquiries can be directed to the corresponding authors.
